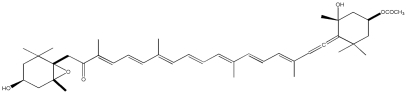# Anti-photoaging and Photoprotective Compounds Derived from Marine Organisms

**DOI:** 10.3390/md8041189

**Published:** 2010-04-08

**Authors:** Ramjee Pallela, Yoon Na-Young, Se-Kwon Kim

**Affiliations:** 1 Department of Chemistry, Pukyong National University, Busan 608-737, Korea; E-Mails: rpallela@gmail.com (R.P.); dbssud@hanmail.net (Y.N.-Y.); 2 Marine Bioprocess Research Center, Pukyong National University, Busan 608-737, Korea

**Keywords:** anti-photoaging, photoprotection, UV irradiation, reactive oxygen species (ROS), matrix metalloproteinases (MMPs)

## Abstract

Marine organisms form a prominent component of the oceanic population, which significantly contribute in the production of cosmeceutical and pharmaceutical molecules with biologically efficient moieties. In addition to the molecules of various biological activities like anti-bacterial, anti-cancerous, anti-inflammatory and anti-oxidative *etc.*, these organisms also produce potential photoprotective or anti-photoaging agents, which are attracting present day researchers. Continuous exposure to UV irradiation (both UV-A and UV-B) leads to the skin cancer and other photoaging complications, which are typically mediated by the reactive oxygen species (ROS), generated in the oxidative pathways. Many of the anti-oxidative and anti-photoaging compounds have been identified previously, which work efficiently against photodamage of the skin. Recently, marine originated photoprotective or anti-photoaging behavior was observed in the methanol extracts of *Corallina pilulifera* (CPM). These extracts were found to exert potent antioxidant activity and protective effect on UV-A-induced oxidative stress in human dermal fibroblast (HDF) cells by protecting DNA and also by inhibiting matrix metalloproteinases (MMPs), a key component in photoaging of the skin due to exposure to UV-A. The present review depicts various other photoprotective compounds from algae and other marine sources for further elaborative research and their probable use in cosmeceutical and pharmaceutical industries.

## 1. Introduction

Human skin is the potential anatomical barrier for pathogens and damage, which acts as an important fence between internal and external environment in the bodily defense. Continuous exposure to UV light leads to numerous complications that are correlated with various pathological consequences of the skin damage and sunburn occurs when exposure to UV light exceeds the protective capacity of an individual’s melanin [1,2]. UV irradiation induces photo-damage of the skin, which is characterized by distinct alterations in the composition of the dermal extracellular matrix (ECM), resulting in wrinkles, laxity, coarseness, mottled pigmentation and histological changes that include increased epidermal thickness and connective tissue alteration [3,4]. Breakdown of a balance of combination between the connective tissue components (biomolecules including collagens, proteoglycans and glycoproteins) leads to the detrimental effects e.g., photoaging in dermal fibroblasts. Skin contains antioxidant defenses, which nullify reactive oxygen species (ROS) including free radicals, but these defenses will be overwhelmed if the dose of UV light is high enough, and this result in free radical damage to cellular components such as proteins, lipids and DNA [5,6]. ROS induced by oxidative stress can ultimately lead to apoptotic or necrotic cell death [7]. Especially, the accumulated ROS plays a critical role in the intrinsic aging and photoaging of human skin *in vivo*, thus suggested to be responsible for various skin cancers and other cutaneous inflammatory disorders [8,9].

Several investigations have revealed that collagen gets degraded in photoaged skin due to the inhibition of collagen synthesis mediated by matrix metalloproteinases (MMPs), which are a family of secreted or transmembrane zinc endopeptidases that are capable of digesting ECM. MMPs are divided into subclasses of collagenases, gelatinases, stromelysins, matrilysins and membrane-type MMPs (MT-MMPs) according to their substrate specificity and domain structure [10]. It is reported that ROS effects the MMP gene expression through signal transduction pathway [11]. Expression of MMPs is usually induced by various extracellular stimuli such as growth factors, cytokines, tumor promoters and UV. Over expression of MMPs has been observed in tissue remodeling, repair and destruction by many extracellular stimuli and among the MMPs, MMP-2 and MMP-9 degrade the ECM and they influence skin wrinkle formation and skin thickness [12]. Previous studies from our lab have identified many MMPIs (MMP inhibitors) including the chitooligosaccharides and other marine extracts [2,13–19].

Photoprotection is a group of mechanisms that nature has developed to minimize the damages that an organism suffers, when exposed to UV radiation. These mechanisms can be controlled or organized by certain organic and inorganic compounds or substances (e.g., melanin) produced by different terrestrial and aquatic sources. A number of photo protective compounds such as scytonemins (exclusively in cyanobacteria), mycosporines (in fungi), mycosporine-like amino acids (MAAs, in cyanobacteria, algae and animals), phenyl propanoids and flavonoids (higher plants), melanins (humans and other animals and even some bacteria), and several other UV-absorbing substances of unknown chemical structures from different organisms have been developed to counteract the photodamage [20,21]. Nearly a decade before, Groniger *et al.* developed a database that contains information on the photoprotective compounds such as MAAs, scytonemin and other unidentified compounds that absorb in the UV range, which are reported in aquatic organisms like cyanobacteria, phytoplankton and macroalgae of diverse habitats at different collection times [22].

The induction of cellular phototoxicity in response to UV radiation has previously been demonstrated in different cell lines [23–25]. Recent research policies have elucidated these cellular photochemical mechanisms and the complex nature of the light-harvesting pigment–protein complexes of microorganisms like algae and fungi and their symbiotic forms in lichens, sponges and corals. The pigments they synthesize and commercial uses of these and other metabolites have been comparatively discussed recently [26]. Microbial compounds in the present era are exerting outstanding potentialities for the benefit of human kind; significantly, the extremophiles are much advantageous to produce novel molecules that are resistant to extremities, for human applications [27,28].

More than 12,000 novel chemicals from marine plants, animals, and microbes have been explored, and a large number of pharmaceutical products, enzymes and biomaterials have been developed until date. Few of the important marine derived photoprotective compounds are presented in Table 1. Potent antioxidative compounds have also been isolated from brown seaweeds; pyropheophytin *a* from *Eisenia bicyclis*, fucoxanthin from *Hijikia fusiformis* and phlorotannin from *Ecklonia stolonifera* [29–31]. Present approaches on marine algal compound isolation and characterization are very attractive because of their potential applications in medicine and pharmaceutical industries. Though the isolation and characterization of the marine molecules from non-algal sources are competitive, the number of algal compounds, especially the photoprotective molecules are high when compared to the other marine sources. Hence, in the present review, we present a detailed latest study on the anti-photoaging or photoprotective compounds from algae and other marine organisms. This will help to improve present research strategies and open future perspectives of understanding the photo-acclimation mechanisms in different marine flora and fauna, which deliver these potent compounds for the beneficial role in the cosmeceutical and pharmaceutical applications.

## 2. Algae

Marine algae have been an important source to produce a variety of secondary metabolites including phenols and polyphenols with unique linkages [32]. From past few decades, marine algae including many of the phytoplanktonic species have been recognized as one of the greatest source of bioactive molecules of significant medicinal and nutritional values. Marine algae produce a wide variety of secondary metabolites possessing many different skeletal types and biological activities [33–35].

### 2.1. Macroalgae

Recently, Ryu *et al.* demonstrated that methanol extracts of alga *Corallina pilulifera* (CPM) possess high phenolic content, which reduces the expression of UV-induced MMP-2 and -9 in human dermal fibroblast (HDF) cells in dose dependent manner, there by attaining the capability of inhibiting free radicals [2]. Another study has been recently undertaken on the photoprotective effect of phlorotannins from *Eckloina cava* against the photo-oxidative stress induced by UV-B radiation *via* DCFH-DA, MTT, comet assay, and microscopic analysis [36]. According to their postulations, among the isolated phlorotannins, dieckol showed prominent inhibitory activity against melanogenesis and effectively reduced UV-B radiation-induced cellular damage, which further indicated that dieckol from *E. Cava* can be used as an effective natural source to make cosmeceutical or pharmaceutical products.

Since a combination of phototherapy using UV-B with topical reagents has been clinically applied to treat hyperproliferative skin disease; sargaquinoic acid and sargachromenol from *Sargassum sagamianum* suggested to be effective therapeutic agents in protection against UV-B [37]. As per these studies, it was concluded that these algal compounds could reduce UV-B induced apoptosis *in vitro* and *in vivo* by the activation of caspases. These same compounds from *S. macrocarpum* were found to have resulted in neurite outgrowth and supporting the survival of neuronal PC12D cell [38]. Later, de la Coba *et al.* documented the cutaneous photoprotective ability of the high UV-absorbing MAAs, Porphyra-334 and shinorine (P-334 + SH), isolated from the red alga *Porphyra rosengurttii*, by *in vivo* procedures on mouse skin [39]. They have also investigated the expression of the heat shock protein HSP70, as a potential biomarker for acute UV damage. In the year 2002, Lyons and O’Brien studied astaxanthin (AST) containing algal extracts, which show modulatory effects on the UV-A irradiated human skin fibroblasts, human melanocytes and human intestinal Caco-2 cells [40]. Their studies indicated that these extracts depicted tremendous alterations in the antioxidants like SOD and GSH, and also showed protective effect against the UV-A induced DNA damage, when used in significant concentrations (equivalent to around 10 μM of AST). It was thus concluded that the algal extracts have a suggestive role as potential antioxidants. Another species of the same alga, *Sargassum siliquastrum* can also produce xanthin compounds and more recently, Heo and Jeon isolated fucoxanthin, a natural carotenoid that exerts a potential photoprotective effect against UV-B induced cell damage [41].

As discussed earlier, skin damage by reactive oxygen species (ROS) leads to the activation of Protein kinase C (PKC), thus increasing the expression of matrix metalloproteinases (MMPs) and degradation of collagen. UVB is known to induce the expressions of MMP1, MMP3, and MMP9 in the normal human epidermis *in vivo* and among them; MMP1 and MMP3 are thought to be the two major contributors to photoaging [42,43]. Hence, the compounds against these photoaging contributors could show greater potential in medical and cosmeceutical approaches.

### 2.2. Microalgae

Algal phytoplanktons have tremendous impact on the sustainability of the marine ecosystem by being a food source to other marine fauna and by possessing the photoprotecting principle to protect the other inhabitants and algal consumers of marine environment. Jeffrey *et al.* have reported the occurrence of many UV-A and UV-B-absorbing compounds in 206 strains of 152 marine microalgal species [44].

Marine plants are majorily (around 90%) occupied by the phytoplanktonic species and nowadays especially, diatoms are attracting the drug science (nanotechnology, optical systems, semiconductor nanolithography and even using diatom valves as vehicles for drug delivery), because of their vast species diversity and distribution all over the world [45]. The diatom, *Thalassiosira weissflogii* is a common species of coastal marine phytoplankton, which might be forming extensive, non-toxic occasional blooms during early summer in the upper layers of the seawater [46]. It suggests that this diatom has the ability to cope up with UV-B indirectly and might be able to develop effective photoprotective strategies upon prolonged exposure to UV-B [47].

Diatoms often dominate the algal blooms during spring and fall at temperate latitudes and in summer in Polar Regions, which show a wide range of response to UV exposure. Centric diatoms seem more UV-resistant than pennate diatoms, after a suitable acclimation period [48]. However, in general, diatoms seem to possess lower chlorophyll-specific concentrations of UV-absorbing pigments than other algal groups [44]. It is very difficult to assess the potential ecological impact on natural phytoplankton, while increase in the in-flux of UV-B radiation resulting from ozone depletion in the atmosphere. One of the main reasons for this difficulty is that UV sensitivity is species specific, which should be considered as a primary criteria for developing various potential anti-photoaging compounds from different sources [47,49–51].

## 3. Other Marine Sources

### 3.1. Fungi

UV-B absorbing mycosporines with photoprotective activity are present day targets from fugal species e.g., lichenized ascomycete, *Collema cristatum* [52]. The pure compound from this source prevented UV-B induced cell destruction in a dose-dependent manner and partially prevented pyrimidine dimer formation. When applied to the skin prior to irradiation, it completely prevented UV-B induced erythema. Kogej *et al.* later identified two different mycosporines and three unidentified UV-absorbing compounds in fungal isolates from hyper-saline waters and polar glacial ice [53].

### 3.2. Lichens

Extensive studies on the secondary metabolites of lichens have led to the isolation of many novel substances, which by number are over 800 [54,55]. These biologically and pharmacologically active compounds comprise aliphatic, cycloaliphatic, aromatic, and terpenic compounds that are similar to those of higher plants. Reactive oxygen species (ROS) and reactive nitrogen species (RNS) production by both UV-A and UV-B contributes to inflammation, immunosuppression, gene mutation and carcinogenesis. Substances that are able to inhibit these reactive species could be used in the prevention of skin cancer, including melanoma, as there is no effective long-term treatment for patients suffering from the advanced stages of this cancer [56–58]. Recently, Russo *et al.* have evaluated the effect of two lichen compounds, sphaerophorin (depside) and pannarin (depsidone) on pBR322 DNA cleavage induced by hydroxyl radicals (^•^OH), and by nitric oxide (NO), and their superoxide anion (O_2_^−^) scavenging capacity [59]. These compounds may be used in the medical field to treat skin cancers.

Rancan *et al.* have analysed the light-filtering power of natural substances like boldine, a major alkaloid found in the leaves and bark of boldo tree *Peumus boldus* (Molina), and other aromatic compounds (usnic acid, 1-chloropannarine, epiphorelic acid I and II, calicin) extracted from Chilean lichens [60]. These compounds have profound UV protection factors, which are similar to that of the reference compounds and their formulations like Nivea sun Spray LSF 5, octylmethoxycinnamate (OMC) and 4-*tert.*-butyl-4′-methoxy dibenzoylmethane (BM-DBM).

### 3.3. Bacteria

Many sunscreen/cosmetic compositions have been discovered from bacteria, which have been adopted for the photoprotection of human skin and/or hair, because of their underlying anti-photoaging principle. More interestingly, bacterial melanin, an active photoprotecting pigment protects against the DNA damage under full UV-B irradiation [61]. The bacterial melanin exhibited excellent protection of bioinsecticide against UV-C and natural solar irradiation, thus arising a question that whether the pigment also has a protective effect on DNA against the full UV spectrum [62]. Holmes *et al.* recently described the bacterial (*Klebsiella aerogenes*) photoprotection through extracellular cadmium sulfide crystallites (CdS), where these semiconductor particles absorb radiation in the UV spectral region [63]. Hence, when *K. aerogenes* produces extracellular CdS material in response to the stressed environments containing cadmium ions, a photoprotective layer is formed.

Bacteria, especially the ones like archaea and other extremophiles species have tremendous implication for the survival in extraterrestrial habitats and are very advantageous in present day astrobiological research for the detection of the protectant biomolecules [27].

### 3.4. Cyanobacteria

Cyanobacteria are phylogenetically a primitive group of Gram-negative prokaryotes that possess higher-plant-type oxygenic photosynthesis [64]. As they are known to have a wide range of habitat, they are supposed to have developed mechanisms leading to adaptations to survive in extreme climates and withstand critical processes such as heat, cold, drought, salinity, nitrogen starvation, photo-oxidation, anaerobiosis, osmotic and UV stress [65]. As a matter of general understanding, it may be believed that cyanobacteria may be fairly tolerant to UV radiation, since their presence and evolution preceded the development of the stratospheric ozone layer [20].

There are a number of adaptation strategies, by which cyanobacteria try to avoid high white light and ultraviolet radiation stress. These adaptations range from migration into habitats of reduced light exposure along with phototactic, photokinetic and photophobic responses [66]; and vertical migration [67]; production of quenching agents such as carotenoids [68] or systems such as superoxide dismutase that neutralize the highly toxic reactive oxygen species produced by UV-B radiation [69]. A number of repair mechanisms such as photoreactivation, light-independent nucleotide excision repair of DNA [70], UV-A/blue-light mediated repair of the photosynthetic apparatus [71] and chromatic adaptation [72] have also been observed. However, the most common protective mechanism is the production of ultraviolet-absorbing substances such as mycosporine-like amino acids (MAAs) and scytonemin [20,73]. Brenowitz and Castenholz established the correlation between UV protection and scytonemin presence under solar irradiance in monospecific population of *Calothrix* sp.; a naturally occurring cyanobacterium [74]. It was shown that high scytonemin content is required for uninhibited photosynthesis under high UV flux.

### 3.5. Marine Animals

Marine animals are largely unexplored for the production of biologically active secondary metabolites. A new marine polypeptide (molecular mass 879) has been recently isolated from the Chinese scallop *Chlamys farreri* and its possible protective role against UV-B induced apoptosis in murine thymocytes has been investigated [75]. This study suggested that the polypeptide was able to avert UV-B induced apoptosis in thymocytes by modulating c-fos and c-jun expression, cytochrome C release, and the consequent activation of caspase-3. UV-A, the longwave (320–400 nm) UV radiation also has a detrimental effect on human skin in terms of photoaging and photodamage. Certain marine compounds have been isolated and have been found to be effective against UV-A induced photodamage. A recent study by Li *et al.* has depicted the role of this polypeptide from *C. farreri* in the inhibition of UVA induced apoptotic pathway in human HaCaT keratinocytes [76]. The molecular mechanisms involved in this protection process were further studied in HaCaT cells radiated by UV-A and it was found that this polypetide significantly reduced the intracellular ROS production, diminished the expression of acid sphingomyelinase (ASMase) and phosphorylated JNK, in a dose-dependent manner [77].

Presence of MAAs has also been reported in the black sea cucumber *Holothuria atra* (Jaeger) and their probable role in photoprotection has been hypothesized. It is believed that MAAs could probably function as broad-spectrum UV absorbers [78]. UV absorbing compounds have also been isolated from the ovaries of scallop *Patinopecten yessoensis* in 2008 by Oyamada *et al.* [79]. It was found that the examined MAAs protected the cells from UV induced cell death and had a protective effect on human cells. It is further expected that these compounds may have potential applications in cosmetics as anti-photoaging/photoprotective agents.

In certain cases, the debate is still on to determine photoprotective compounds and their sure role in photoprotection in marine organisms. One such example is the study on spawn of the sea hare *Aplysia dactylomela* by Carefoot *et al.*, where the role of MAAs as protective sunscreens was investigated, but the significant mechanisms and commercial prospectives are still open for discussion [80].

## 4. Conclusions

UV irradiation is a present day concern, as the impact of global warming and other climatic catastrophic factors are severely effecting the biological population. Hence, photoprotection is a major biological concern with respect to the source of natural bioactive molecules that have the anti-photoaging effect and especially, the safer marine sources have been identified in past few decades. The advances in molecular biology and culturing technologies are bridging the gap between the challenges pertaining to the exploitation of marine environment as a potential source of natural protective compounds. As presented in the current review, various compounds which support potentially the photoprotective mechanisms of strong cosmeceutical and pharmaceutical value, have already been isolated from different marine sources like algae, fungi, lichens, bacteria, phytoplanktons, animals and plants. Several marine based pharmaceuticals are under active commercial development, as the ecosystem health is high on the public’s list of concerns, and hence, aquaculture is providing an ever-greater proportion of the seafood in developed countries. An extensive research still has a major role to play to uncover the enormousity of the vast marine sources in terms of photoprotective compounds that promote DNA protection by reducing oxidative stress after UV exposure. This would develop a proper understanding of the *in-situ* and *ex-situ* approaches of marine floral and faunal culture for the development of novel photoprotective molecules, by considering the global warming and other catastrophic causes of ozone destruction.

In the light of present review, it is worthwhile to state that anti-photoaging and photoprotective natural products research from such deceptively simple marine organisms will pave a way for newer and novel products, which find immense use in the pharmaceutical and cosmeceutical industry.

## Figures and Tables

**Table 1:** List of a few photoprotective and anti-photoaging compounds from marine organisms.

No.	Compound	Structure
1.	Eckol	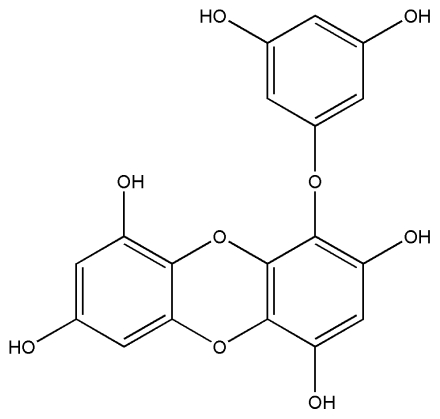
2.	Mycosporine methylamine-serine	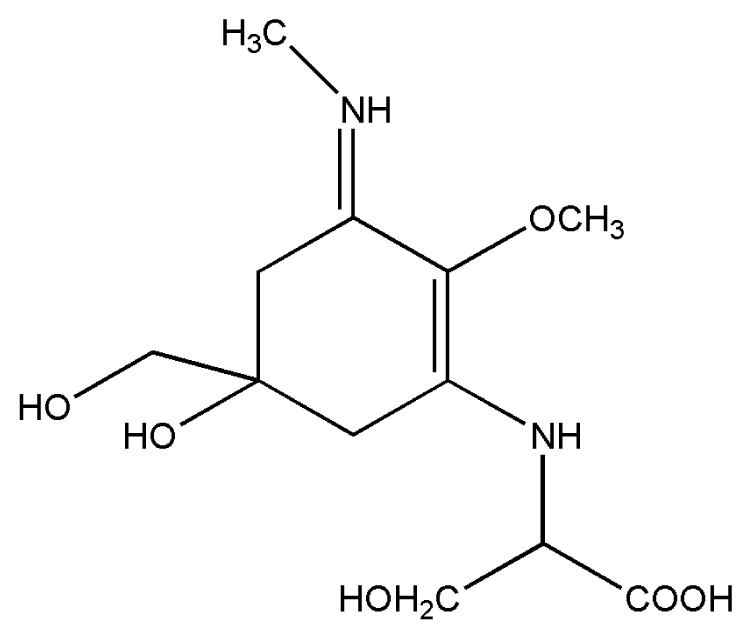
3.	Mycosporine-glycine	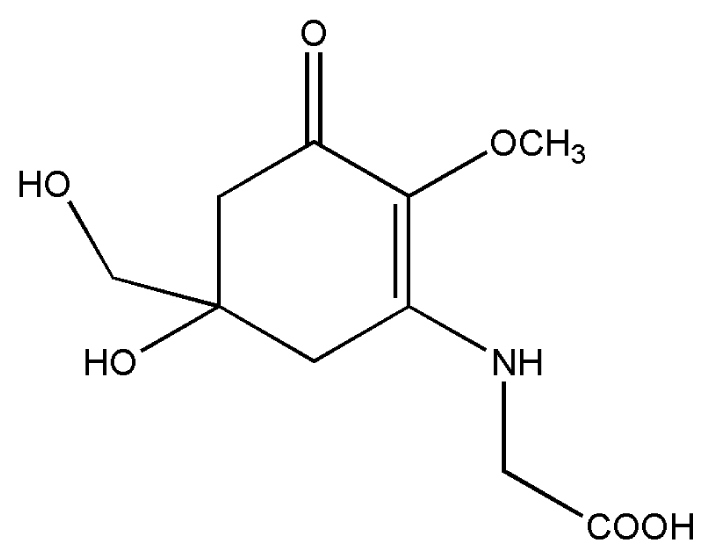
4.	Palythene	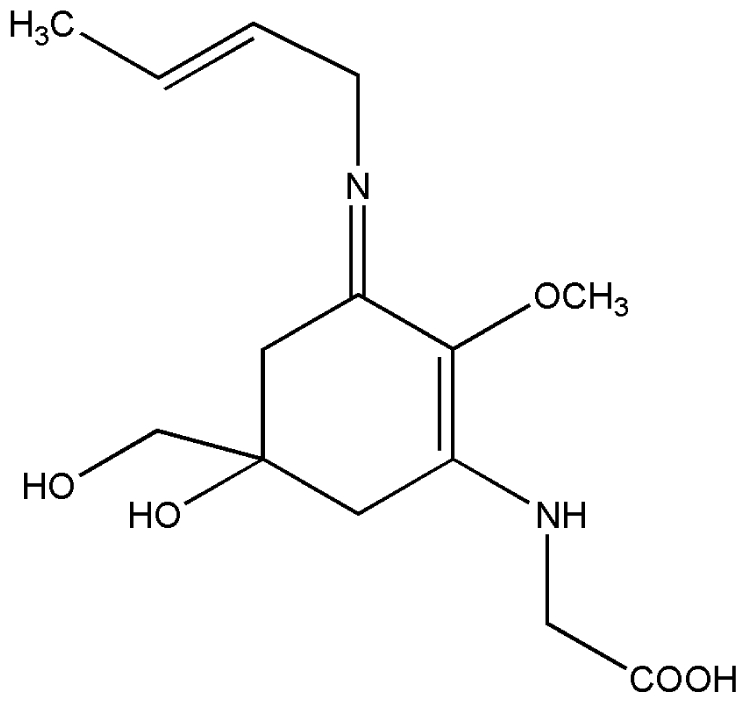
5.	Shinorine	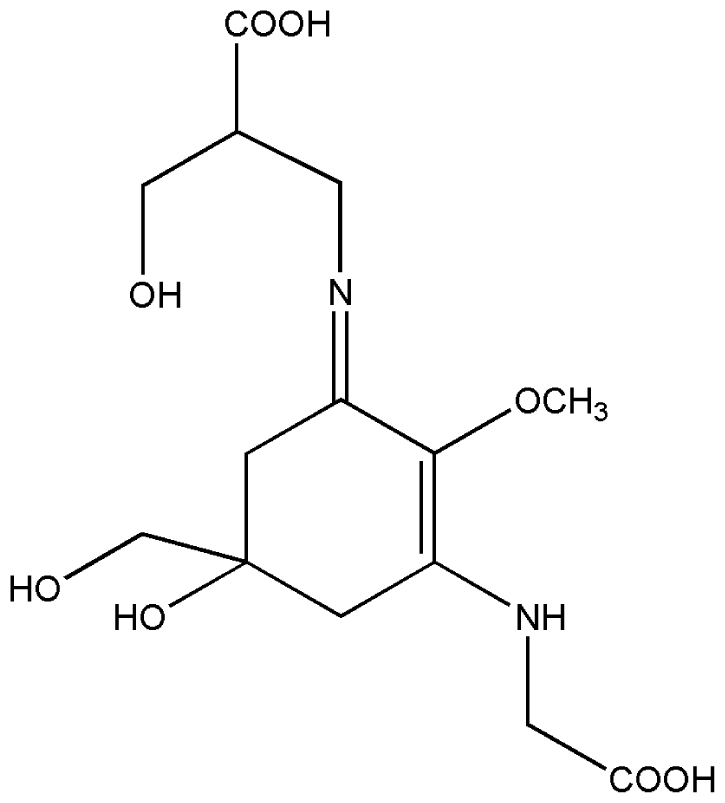
6.	Porphyra-334	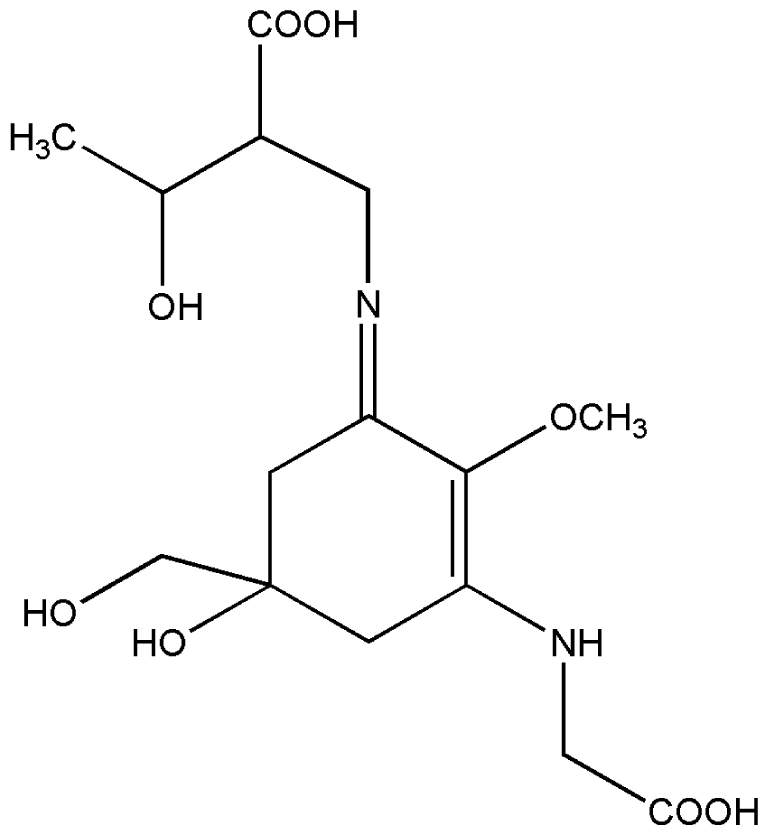
7.	Scytonemin	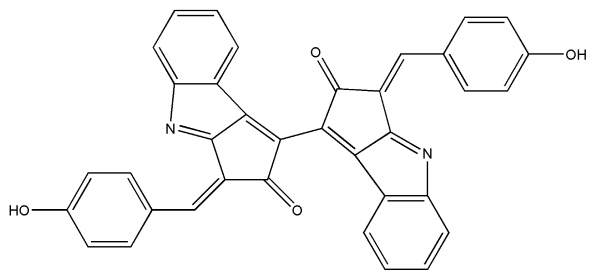
8.	Sargaquinoic acid	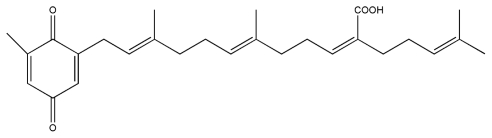
9.	Sargachromenol	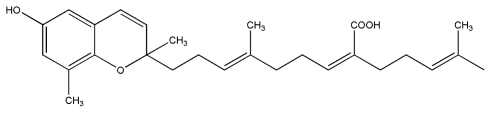
10.	Fucoxanthin